# Periodontal Disease Diagnosis by a Chemically Etched Single-Mode Fiber-Optic Biosensor for Label-Free Detection of Matrix Metalloproteinase-8 (MMP-8)

**DOI:** 10.3390/bios16090491

**Published:** 2026-09-03

**Authors:** Rigoberto Tovar, Sarkis Sozkes, Marzhan Sypabekova

**Affiliations:** 1 Department of Bioengineering, Civil Engineering, and Environmental Engineering, U. A. Whitaker College of Engineering, Florida Gulf Coast University, 10501 FGCU Boulevard South, Fort Myers, FL 33965, USA; 2 Department of Biomaterials, Biomedical Engineering Corlu Faculty, Tekirdag Namik Kemal University, Yılmaz Alpaslan Cd. No:25, 59850 Tekirdag, Turkey

**Keywords:** optical fiber biosensor, evanescent wave, etched single-mode fiber, MMP-8, periodontal disease, label-free detection, spiked saliva, point-of-care diagnostics

## Abstract

A miniature label-free biosensor based on a chemically etched single-mode optical fiber (SMF) is reported for the detection of matrix metalloproteinase-8 (MMP-8), a salivary biomarker of active periodontitis with a clinical decision threshold of 20 ng/mL. Fibers etched in 48% hydrofluoric acid to a waist diameter of 16.0 ± 1.4 µm gave a mean refractive index (RI) sensitivity of 376.8%/RIU and an RI limit of detection (LOD) of 5.9 × 10^−4^ RIU. Fibers were tested with MMP-8 spiked into phosphate-buffered saline (PBS) and into saliva over 0–200 ng/mL using a post-rinse protocol with per-fiber matrix subtraction. Dose–responses followed a Langmuir isotherm (Kd = 7.1 ng/mL in PBS, 12.7 ng/mL in saliva), with cohort LODs of 0.043 and 0.52 ng/mL, both well below the threshold. MMP-9 (100 ng/mL) and human serum albumin (1 mg/mL) gave negligible responses (≤2.7%, versus 68.2% for MMP-8 at 100 ng/mL); antibody immobilization was confirmed by confocal immunofluorescence. A commercial sandwich ELISA on the same spike series gave a matched-matrix LOD of 41.1 ng/mL, nearly two orders of magnitude higher. This performance requires no metal coating, nanostructuring, label, or signal amplification, only a single wet-etching step on stock telecommunications fiber.

## 1. Introduction

Periodontitis is a chronic inflammatory disease of the tooth-supporting structures that affects approximately 47% of adults over the age of 30 in the United States, with severe forms reported in 8–10% of the global population [1,2]. It is the leading cause of adult tooth loss and has been epidemiologically linked to systemic conditions including cardiovascular disease, diabetes, and adverse pregnancy outcomes [3,4]. Routine clinical diagnosis is based on probing pocket depth, clinical attachment level, and radiographic bone loss, measurements that are subjective, retrospective, and require trained clinicians [5].

Among the salivary biomarkers that reflect the biochemical processes underlying periodontal tissue destruction, MMP-8, a collagen-degrading enzyme released primarily by neutrophils during the inflammatory response, is the most extensively validated single biomarker of active periodontitis [6,7]. Salivary MMP-8 levels below 20 ng/mL are generally associated with periodontal health, while concentrations exceeding 20 ng/mL indicate active disease, with advanced periodontitis often reaching 100–200 ng/mL or higher [8,9]. The current quantitative gold standard for MMP-8 measurement is the sandwich enzyme-linked immunosorbent assay (ELISA), which requires laboratory infrastructure and 2–4 h per run [10]; its sensitivity is further degraded in complex biological fluids such as saliva [11]. This has motivated the development of rapid, portable platforms for chairside screening.

Optical fiber biosensors have drawn extensive interest over the last two decades due to their compact size, flexibility, biocompatibility, immunity to electromagnetic interference, and capability for real-time label-free measurement [12,13]. A common detection principle exploits the evanescent field of a guided mode by physically modifying the fiber so that part of the optical power propagates outside the core and interacts with the analyte [14]. Such modifications include chemical etching [14,15], tapering [16], and grating photo-inscription [17]. Among these, chemical etching with HF is particularly attractive because it does not require a heat source, produces a uniform-diameter waist, and is compatible with high-volume manufacturing [18].

A small number of biosensors have been reported for MMP-8 specifically. Guida et al. [19] demonstrated a surface plasmon resonance sensor on a D-shaped plastic optical fiber, reporting an LOD of 1.76 ng/mL in buffer and 9.9 ng/mL in saliva; the probe requires side-polishing of the fiber core, a 1 µm photoresist buffer layer, and a 60 nm sputtered gold nanofilm. Taylor et al. [20] reported a surface acoustic wave immunosensor built on a piezoelectric quartz chip with gold interdigitated electrodes, with an estimated LOD of 62.5 ng/mL over a 0–1000 ng/mL working range and validation in unstimulated clinical saliva; the assay uses a sandwich format requiring a secondary anti-MMP-8 detection antibody. Other platforms reach lower limits at the cost of added complexity: the voltammetric immunosensor of Tortolini et al. [21] achieved 1.0 ng/mL in buffer using gold-nanosphere-decorated graphene screen-printed electrodes, and the Immuno-CRISPR/Cas12a assay of Wu et al. [22] reached fg/mL levels in saliva through gold-nanoparticle-coupled enzymatic signal amplification (Table 1).

The sensor reported here reaches sub-ng/mL detection limits in both buffer and spiked saliva and does so with substantially less fabrication and assay complexity than any previously reported MMP-8 platform. Transduction relies solely on the evanescent field of a chemically etched standard telecommunications SMF, with no metal or plasmonic coating, no nanomaterial or nanostructuring, no grating photo-inscription, and no polishing or lithographic buffer layer; the entire sensing element is produced by a single wet-etching step on stock fiber. Among the routes to thinned-fiber evanescent sensors, HF etching is the lowest cost, requiring no laser, filament, arc, or splicing equipment [23]. Detection is single-step and label free, requiring no secondary detection antibody, no redox mediator, and no enzymatic amplification, so quantification comes from one transmission spectrum acquired with a broadband source and a compact spectrometer.

**Table 1:** Comparison of MMP-8 detection methods.

Method	Sensing Principle	Range (ng/mL)	LOD and Sample Matrix	Nano Structuring and Label-Free	Reference
SPR on D-shaped plastic fiber	SPR	0.1–20	Buffer 1.76 (ng/mL) Saliva 9.9 (ng/mL) (recombinant spike series)	No	Guida et al. [19]
SAW immunosensor	Surface acoustic wave	0–1000	Buffer 62.5 (ng/mL) Saliva (177 subjects: periodontitis, gingivitis, healthy)	No	Taylor et al. [20]
Voltammetric immunosensor	Electrochemical	2.5–300	Buffer 1.0 (ng/mL) Saliva (10 volunteers)	No	Tortolini et al. [21]
Immuno-CRISPR/Cas12a assay	Fluorescence, Cas12a trans-cleavage	Not stated	fg/mL Saliva (23 saliva samples)	No	Wu et al. [22]
PerioSafe^®^/ORALyzer^®^	Lateral flow IA	semi-quant.	~20 (ng/mL) Mouthrinse/GCF	No	Sorsa et al. [24]
Commercial ELISA	Sandwich ELISA	0.063–4.0	Proprietary assay diluent (buffer) 0.063 ng/mL	No	Leppilahti et al. [10]
Etched SMF	Evanescent wave	0–200	PBS 0.043 (ng/mL) Saliva 0.52 (ng/mL) (recombinant spike series)	Yes	This work

## 2. Materials and Methods

### 2.1. Reagents

Corning^®^ SMF-28e+ Single mode Optical Fiber 9/125/250 µm was purchased from Fiber-Mart, Phoenix, AZ, USA. Sulfuric acid (H_2_SO_4_, 96%), hydrogen peroxide (H_2_O_2_, 30%), (3-aminopropyl)triethoxysilane (APTES, 99%), glutaraldehyde (25%), bovine serum albumin (BSA, ≥98%), sucrose, and phosphate-buffered saline (PBS) tablets were purchased from Sigma-Aldrich, St. Louis, MO, USA. Monoclonal anti-human MMP-8 antibody (MAB9081-SP) and recombinant human MMP-8 (908MP010) were purchased from R&D Systems, Minneapolis, MN, USA. Recombinant human MMP-9 (RP-75653), recombinant human IL-1β (200-01B-10UG), human serum albumin (HSA, A9511), Alexa Fluor 488 goat anti-mouse IgG (A28175), saliva (NC1873815), and the Human MMP-8 ELISA kit (EHMMP8) were obtained from Thermo Fisher Scientific, Waltham, MA, USA

### 2.2. Optical Setup and Interrogation

**Figure 1:**
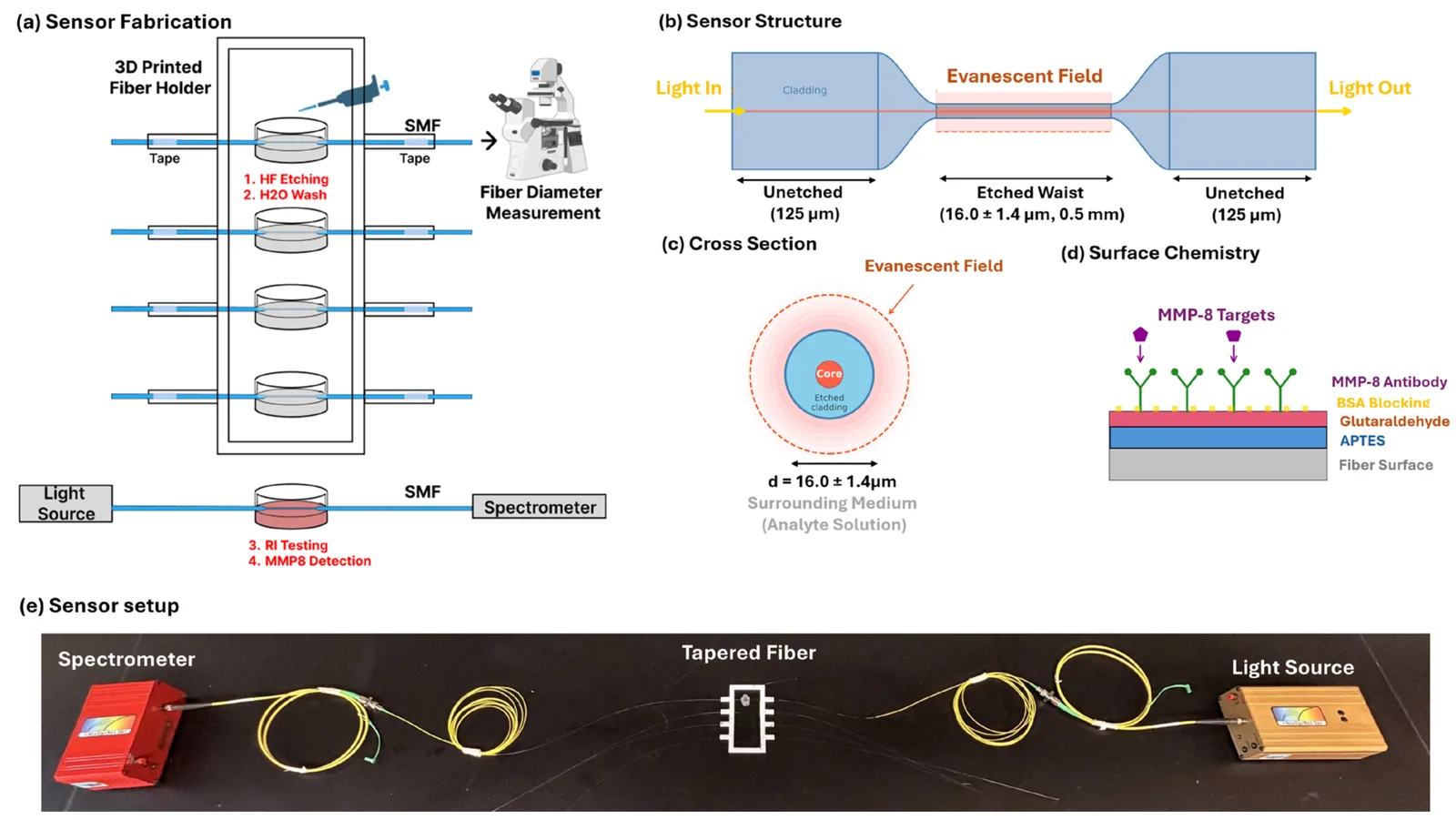
Etched optical fiber MMP-8 biosensor fabrication and design. (**a**) Fabrication workflow showing HF etching of the SMF using a 3D-printed fiber holder, followed by diameter measurement, RI calibration, and MMP-8 detection. (**b**) Sensor structure showing the etched-waist region, where the evanescent field interacts with the surrounding analyte solution. (**c**) Cross-section of the etched fiber with reduced cladding diameter. (**d**) Surface functionalization strategy using APTES, glutaraldehyde, BSA blocking, and anti-MMP-8 antibody for target capture. (**e**) A photograph of the physical sensor and measurement configuration (the etched optical fiber mounted in the laser-cut holder, connected between the broadband source and the spectrometer).

The optical interrogation system consisted of a broadband deuterium-halogen source (StellarNet SL5, 190–2500 nm) coupled into one end of the sensing fiber and a thermoelectrically cooled InGaAs spectrometer (StellarNet DWARF-Star NIR) at the other end (Figure 1e). The spectrometer covered 1000–1700 nm with a 1024-element InGaAs photodiode array (~0.68 nm/pixel), 16-bit analog-to-digital conversion, and a manufacturer-specified signal-to-noise ratio of 4000:1. Spectra were acquired with integration times of 1000–1500 ms. SpectraWiz, version 7, StellarNet Inc., Tampa, FL, USA (StellarNet) software was used for off-line analysis.

### 2.3. Fiber Etching and RI Calibration

A 1 cm midpoint section of SMF-28e was stripped of its polymer jacket using a mechanical stripper and cleaned with isopropanol. A 0.5 mm stripped section was immersed in approximately 125 µL of 48% HF in an Eppendorf tube placed under a chemical fume hood at room temperature. The HF solution was refreshed twice every 25 min. The total etching time was 50 min. After etching, the fiber was rinsed three times with deionized (DI) water to halt the reaction. The etched diameter of each fiber was measured by optical microscopy (Leica DM4000 B, 20×/0.50 objective) and was 16.0 ± 1.4 µm (mean ± SD, n = 10) for the fibers used in this work (Figure 1a–c).

The RI sensitivity of each etched fiber was characterized prior to functionalization using a sucrose calibration series at 0, 0.39, 0.781, 1.56, 3.125, 6.25, 12.5, and 25% (*w*/*v*). The corresponding refractive index of each solution was computed from the Barber polynomial [25], giving an RI range of 1.333–1.372 RIU. For each calibration point, the fiber was fully immersed in the solution, allowed to stabilize for 10 min, and the transmitted spectrum was recorded. A custom laser cut multi-fiber holder made from medium white acrylic was used to maintain the fiber in a fixed position throughout etching, silanization, and optical measurements, ensuring consistent alignment across all processing steps (Figure 1e).

### 2.4. Fiber Surface Functionalization

The etched fibers were functionalized with anti-MMP-8 antibody following silanization protocol adapted from our previous work [14,26]. Briefly, the etched region of the fiber was cleaned with freshly prepared piranha solution (3:1 H_2_SO_4_:H_2_O_2_) for 5 min and rinsed thoroughly with DI water. The cleaned fiber, mounted in the holder, was dried using a nitrogen gun. The plasma-treated fiber was then incubated in 1% (*v*/*v*) APTES in absolute ethanol for 30 min and cured at 100 °C in an oven (Thermolyne Oven, Thermo Scientific) for 30 min to form a stable aminosilane monolayer. The silanized fiber was incubated in 2.5% (*v*/*v*) glutaraldehyde in 10 mM PBS (pH 7.4) for 30 min to expose free aldehyde groups, then rinsed with PBS. Anti-MMP-8 antibody (8.35 µg/mL) was applied to the activated surface and allowed to react for 1 h to form covalent linkages between the surface aldehydes and the lysine residues of the antibody. After PBS rinsing, the fiber surface was blocked with 1% BSA in PBS for 30 min to minimize non-specific adsorption (Figure 1d).

### 2.5. Antibody-Immobilization Verification by Confocal Immunofluorescence

Antibody immobilization and accessibility were verified by confocal microscopy using Alexa Fluor 488 goat anti-mouse IgG secondary antibody. Four spare etched fibers were prepared in parallel: F1, fully functionalized with Alexa secondary antibody as the positive control; F2, prepared without primary anti-MMP-8 antibody but incubated with Alexa secondary antibody to assess nonspecific secondary antibody binding to the silane/glutaraldehyde/BSA surface; F3, fully functionalized without Alexa secondary antibody to assess fiber autofluorescence; and F4, piranha-cleaned bare silica incubated directly with Alexa secondary antibody to assess nonspecific adsorption to the glass surface. Alexa Fluor 488 secondary antibody was applied to F1, F2, and F4 at 5 µg/mL in PBS, while F3 received PBS only, and all fibers were incubated for 1 h at room temperature in the dark followed by three PBS washes. Each fiber was mounted on a clean glass bottom culture dish and imaged using a confocal fluorescence microscope (FV1000, Olympus, Tokyo, Japan) through a FITC/GFP channel with excitation at 488 nm and emission collected at 530 nm, using the 20× objective, 50% laser power, 7× gain, 1000 v HV, and 20% Offset settings for all fibers. Fiber regions of interest were defined from the DAPI channel, and Alexa green mean intensity was measured within each DAPI-guided fiber ROI after subtraction of an adjacent off-fiber local background ROI (Figure S1).

### 2.6. MMP-8 Detection in PBS

After functionalization, three fibers (n = 3) were rinsed with PBS and a baseline transmission spectrum was acquired with the etched region immersed in PBS, taken as the reference (0 ng/mL). Recombinant human MMP-8 was then applied sequentially at 6.25, 12.5, 25, 50, 100, and 200 ng/mL in PBS, in order from low to high concentration. At each step, the fiber was incubated in the MMP-8 solution for 15 min, rinsed with PBS, and a spectrum was acquired in PBS. This protocol covered the clinically relevant range from below the healthy/diseased threshold (20 ng/mL) through advanced periodontitis (>100 ng/mL). Because antibody–antigen binding is effectively irreversible on the timescale of a PBS rinse [27,28], no regeneration of the surface occurs or is claimed between steps: the rinse removes loosely and non-specifically bound material, and each post-rinse spectrum reports the equilibrium cumulative surface coverage produced by the total MMP-8 exposure up to that step. The dose–response is therefore a cumulative-exposure calibration, a standard format for titrating a single antibody-coated sensor [17,19,29], and the Langmuir fit is performed against the cumulative concentration. The sensor is conceived as a single-use disposable, so surface regeneration is not part of the intended workflow.

### 2.7. MMP-8 Detection in Spiked Saliva

Functionalized fibers (n = 3) were tested in saliva spiked with recombinant human MMP-8 at 0, 10, 25, 50, 100 and 200 ng/mL. Each fiber was equilibrated in PBS and a baseline spectrum was recorded (PBS-baseline reference), incubated in the spiked saliva for 15 min, rinsed with PBS, and re-measured in PBS; this post-rinse spectrum was used for quantification. A per-fiber matrix subtraction was then applied, the unspiked-saliva (0 ng/mL) response was subtracted from every spiked point, so that 0 ng/mL is set to 0% and the signal reflects only the added MMP-8 spike above endogenous MMP-8 and non-specific binding.

### 2.8. Specificity Testing Against Non-Target Proteins

The analytical specificity of the functionalized sensor was evaluated against three non-target proteins, each tested on three independently functionalized fibers (n = 3 per analyte): human MMP-9 (a structurally related collagenase of the same Zn^2+^-metalloprotease family) at 100 ng/mL in PBS, human IL-1β (a co-occurring inflammatory cytokine elevated in periodontitis) at 100 ng/mL in PBS, and human serum albumin (HSA, the most abundant interfering protein in oral fluid) at 1 mg/mL in PBS. The challenge concentration for the purified proteins (100 ng/mL) was matched to the high end of the MMP-8 dose–response; HSA was challenged at 1 mg/mL to represent a conservative upper bound of oral-fluid albumin. Each functionalized fiber was equilibrated in PBS and a baseline transmission time series was acquired, then incubated in the non-target solution for 15 min, rinsed with PBS 3x, and re-measured in PBS under identical settings (post-rinse spectrum).

### 2.9. ELISA Cross-Validation in Spiked Saliva

The same MMP-8/saliva spike series (0, 10, 25, 50, 100 ng/mL spiked into saliva) was assayed in triplicate on the Human MMP-8 ELISA kit per the manufacturer’s protocol. Samples were diluted with 1× Assay Diluent B prior to loading. Triplicate well absorbances were averaged and the mean OD_450_ values were fit to a four-parameter logistic (4PL) model as recommended in the kit manual. The kit-defined limit of detection (mean(blank) + 2 × SD(blank) read in dose from the 4PL curve) was computed.

### 2.10. Data Analysis

For each fiber, the normalized intensity change was computed as

(1) $$\Delta I/I_{0}\left(C\right)=\left[{\left\langle I_{C}\left(\lambda \right)\right\rangle }_{\left(\lambda \pm 5\right)}-{\left\langle I_{0}\left(\lambda \right)\right\rangle }_{\left(\lambda \pm 5\right)}\right]/{\left\langle I_{0}\left(\lambda \right)\right\rangle }_{\left(\lambda \pm 5\right)}$$

where ⟨·⟩ denotes the mean intensity over a ±5 nm window centered on the sensing wavelength λ, C is the analyte concentration, and I_0_ is the baseline spectrum. The value of λ was selected per fiber by scanning candidate centers across the sensing band (985–1400 nm, 2 nm steps) and choosing the value that maximized the regression R^2^. The RI calibration was fit with a linear model, y=a×n+b, where n is the refractive index and a is the sensitivity. The MMP-8 dose–responses in PBS and in spiked saliva were fit with a Langmuir isotherm,

(2) $$y\left(C\right)=\left(Imax\cdot C\right)/\left(Kd+C\right)$$

where I_max is the saturation response and K_d the apparent dissociation constant. LOD followed the IUPAC 3σ rule [30]: $LOD=\left(3\times \sigma \right)÷\left|a\right|$ for the linear RI fit, and $LOD=\left(K_{d}\times 3\times \sigma \right)÷\left(I_{m}ax-3\times \sigma \right)$ for the Langmuir fits. Here, σ is the temporal standard deviation of the sensor baseline, obtained from 10 min time-series spectra acquired in the matched matrix, DI water for the RI calibration, PBS and saliva for the respective dose–responses. For presentation, each fiber’s response was normalized to its own saturation amplitude, placing all curves on a common 0–100% scale. Cohort responses are the mean ± standard error of the mean (SEM) of the three per-fiber normalized responses (n = 3 fibers), fit with the same regression form and with cohort σ taken as the mean of the per-fiber σ values. Confocal fluorescence (Alexa-488) was quantified as the mean intensity in a ±6 px band along the fiber centerline, located from the reflectance channel, minus the mean of parallel background bands 14–34 px to either side; values are reported in relative units (a.u.).

Because the etched-waist transmission arises from multimode interference, the sensitivity of the transmitted intensity to surface binding is strongly wavelength dependent, and the wavelength of maximum sensitivity differs between fibers with different waist diameters; the per-fiber scan locates this physical optimum. Since selecting the wavelength on the same data that are subsequently fitted can in principle inflate the fit and lower the LOD, the analysis was also repeated with the sensing wavelength predefined from each fiber’s pre-functionalization refractive-index calibration (Section 3.2).

## 3. Results and Discussion

### 3.1. Etching of the Fiber and Refractive Index Calibration

**Figure 2:**
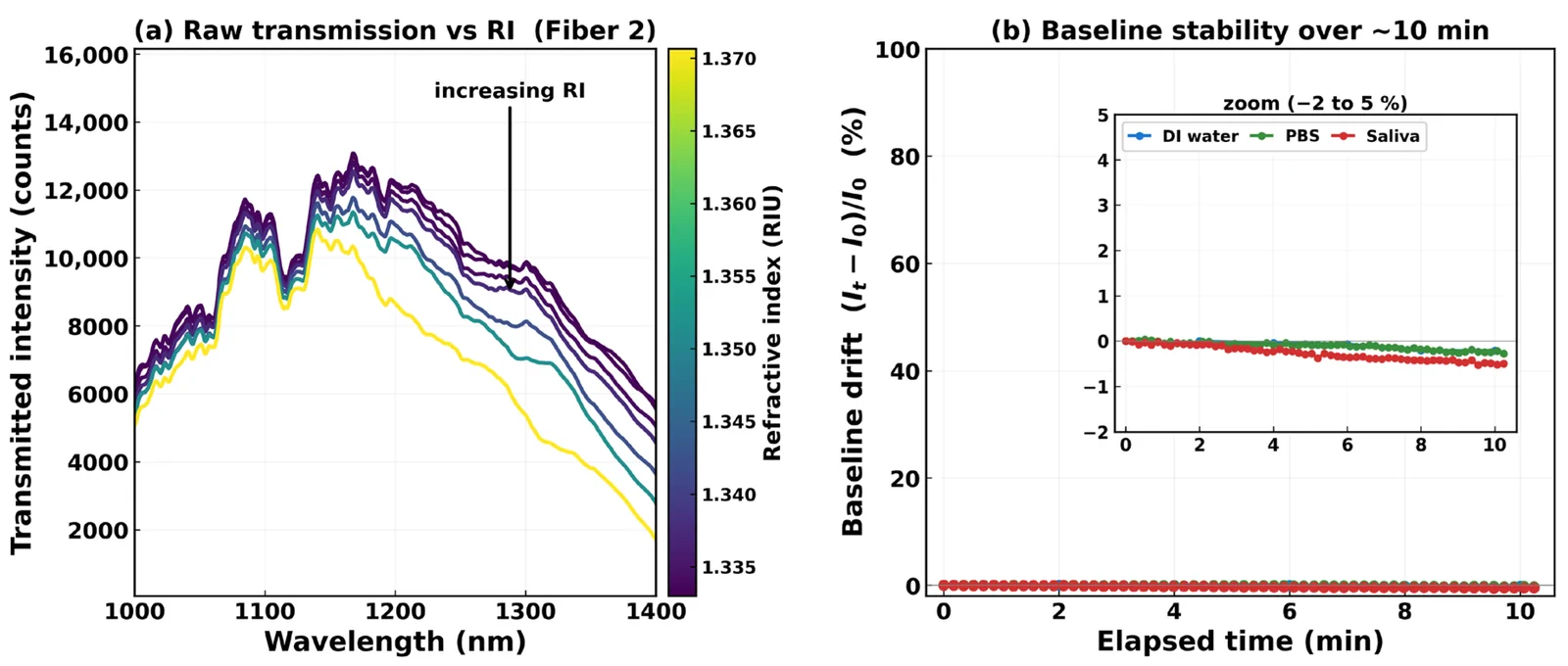
Optical response and baseline stability of the etched fiber sensor. (**a**) Raw transmission spectra of Fiber 2 (1000–1400 nm) measured in solutions of increasing RI (colorbar, 1.333–1.371 RIU); the transmitted intensity decreases with increasing RI (arrow), the response read as the normalized intensity change ΔI/I_0_ at the sensing wavelength. (**b**) Baseline stability of the sensor in DI water, PBS, and saliva over ~10 min, reported as normalized intensity drift (I_t_ − I_0_)/I_0_; all three media drift by less than 1%. The inset shows the same data on an expanded scale (−2 to 5%).

Chemical etching thins the cladding of the SMF at the sensing region, so that the evanescent field of the guided mode extends into the surrounding medium and the transmitted light becomes sensitive to the external refractive index. The transmission spectrum of the etched fiber in the 1000–1400 nm window shows a broad near-infrared envelope with weak interference fringes (Figure 2a, Fiber 2 shown as an example); the fringes arise from the abrupt diameter change at the transition between the unetched and etched regions, which excites and re-couples cladding modes [16,31]. At the operating diameter of approximately 16 µm, the evanescent field at the etched waist overlaps the surrounding medium, so a change in the external RI modulates the transmitted intensity; we read this response as the normalized intensity change ΔI/I_0_. The sensor baseline in DI water, PBS, and saliva was stable over the ~10-min measurement window, drifting by less than 1% in all three media (Figure 2b).

**Figure 3:**
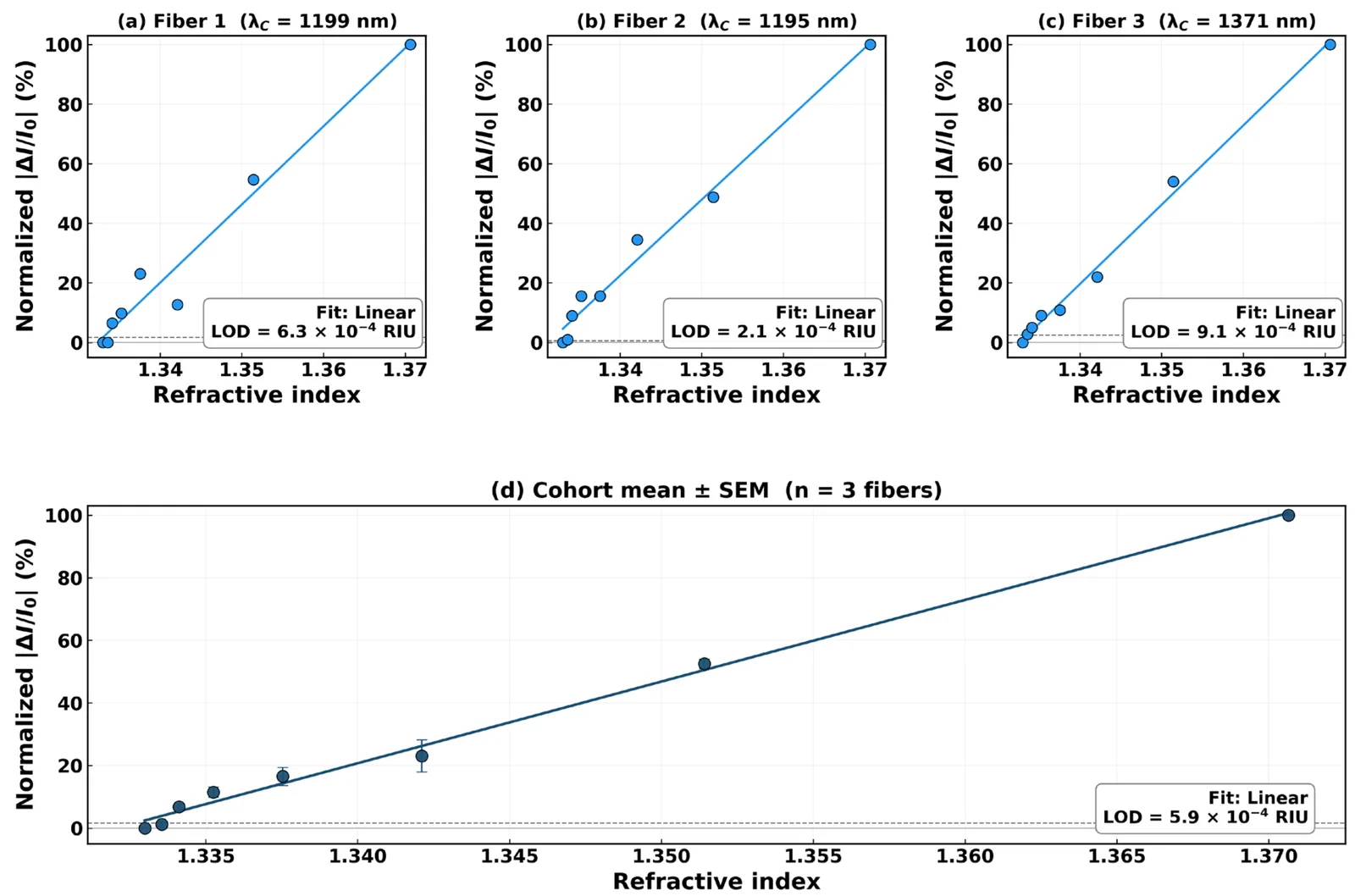
Refractive-index calibration of etched-fiber sensors. (**a**–**c**) Normalized intensity response of three individual etched fibers to increasing RI at their selected sensing wavelengths (1199 nm, 1195 nm, and 1371 nm); solid lines are linear fits used to obtain the per-fiber RI sensitivity and LOD, and the dashed line marks the 3σ detection threshold. (**d**) Cohort calibration curve (mean ± SEM, n = 3 fibers) with a linear fit, giving a cohort R^2^ = 0.994 and an overall LOD of 5.9 × 10^−4^ RIU.

The etched fibers were calibrated against a sucrose RI series spanning 1.333–1.371 RIU. The normalized intensity change at the optimized sensing wavelength was a linear function of the surrounding RI for all three fibers (Figure 3a–c). Per-fiber sensitivities ranged from 165.5 to 701.5%/RIU (mean 376.8%/RIU) and per-fiber LODs from 2.1 × 10^−4^ to 9.1 × 10^−4^ RIU, with all per-fiber R^2^ > 0.96. The cohort calibration (mean ± SEM, n = 3) was linear (R^2^ = 0.994) with an LOD of 5.9 × 10^−4^ RIU (Figure 3d); LODs use the measured DI-water baseline noise as the 3σ detection threshold (Section 2.10). The spread in absolute sensitivity across fibers reflects the diameter-dependent evanescent-field overlap of the etched waist, whereas the consistency of the response shape (per-fiber R^2^ > 0.96, cohort R^2^ = 0.994) indicates a reproducible transduction mechanism. The achieved RI sensitivity is comparable to or better than previously reported HF-etched SMF sensors of similar diameter [14,15,26] and is sufficient to detect the RI changes associated with protein binding at the fiber surface (Δn ~ 10^−3^–10^−2^) [32].

### 3.2. MMP-8 Detection in PBS

**Figure 4:**
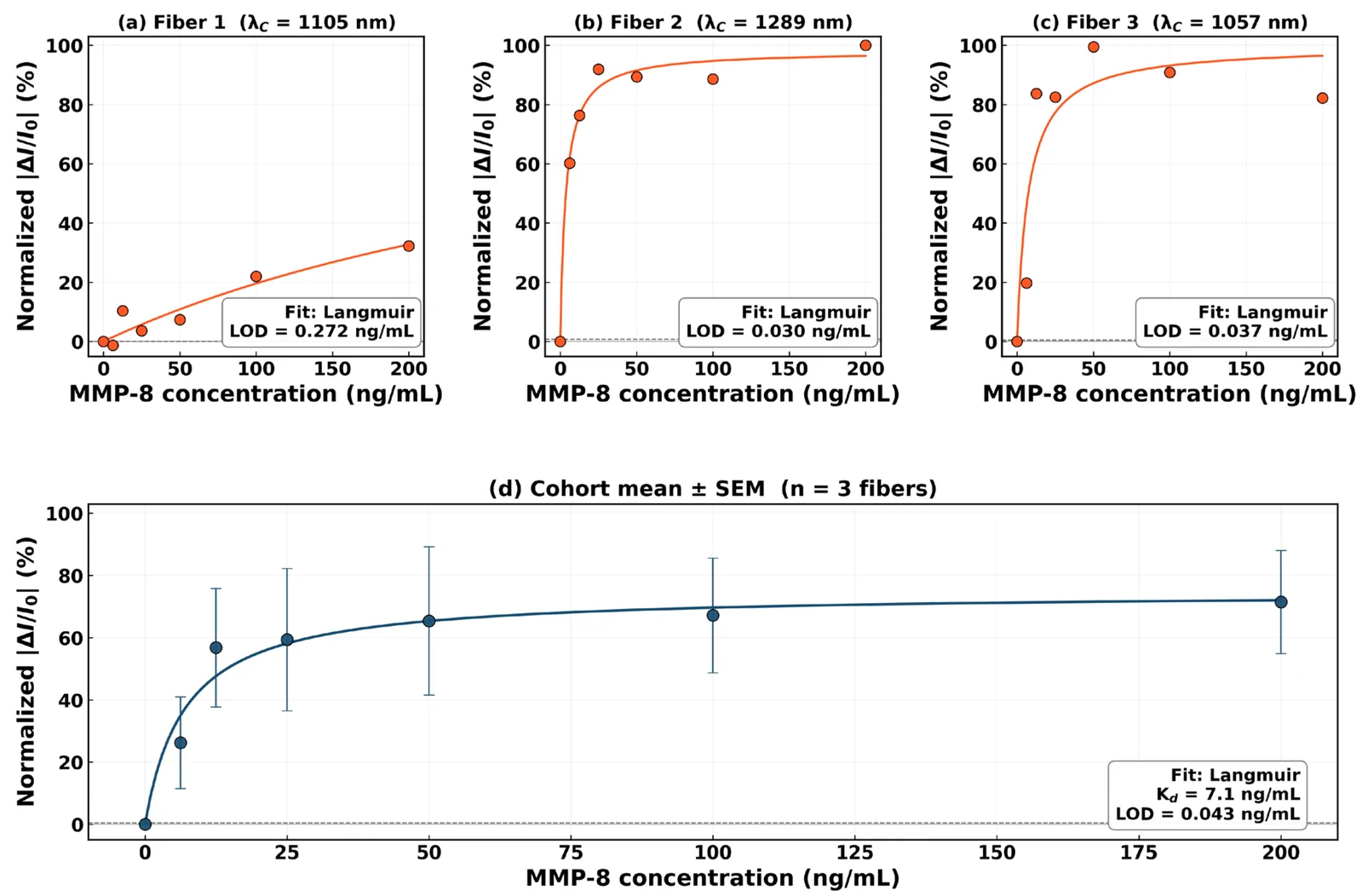
MMP-8 detection in PBS using etched-fiber sensors. (**a**–**c**) Normalized intensity response of three individual antibody-functionalized fibers to increasing MMP-8 concentration in PBS at selected sensing wavelengths of 1105, 1289, and 1057 nm; responses were fitted with a Langmuir binding model, giving individual LODs of 0.272, 0.030, and 0.037 ng/mL, respectively. The dashed line marks the 3σ detection threshold. (**d**) Cohort response (mean ± SEM, n = 3 fibers) with a Langmuir fit yielding K_d = 7.1 ng/mL and an overall LOD of 0.043 ng/mL.

The functionalized fibers were tested for the detection of MMP-8 in PBS over 0–200 ng/mL. The normalized intensity change at the optimized sensing wavelength increased monotonically with MMP-8 concentration and approached saturation at the higher concentrations, consistent with specific antibody–antigen binding described by a Langmuir isotherm (Figure 4). The cohort dose–response (mean ± SEM, n = 3) was fit to the Langmuir model, yielding dissociation constant K_d = 7.1 ng/mL. The cohort LOD, computed via the 3σ rule with the threshold set by the measured PBS baseline noise, was 0.043 ng/mL, more than two orders of magnitude below the 20 ng/mL clinical threshold for periodontitis. Per-fiber LODs ranged from 0.030 to 0.272 ng/mL across the three fibers. The approach to saturation with increasing concentration is characteristic of specific antibody–antigen binding and supports the conclusion that the observed signal change is due to specific MMP-8 capture rather than non-specific adsorption. The cohort K_d value of 7.1 ng/mL falls within the published affinity range for monoclonal anti-MMP-8 antibodies (1–100 ng/mL) [6] and is consistent with a specific recognition event at the fiber surface. The three fibers differed substantially in their individual response: the per-fiber apparent K_d spanned 3.6–411 ng/mL, the optimized sensing wavelength 1057–1289 nm, and the LOD 0.030–0.272 ng/mL, with Fibers 2 and 3 reaching saturation within the tested range while Fiber 1 remained in its near-linear regime. This spread most likely originates from fiber-to-fiber differences in the etched-waist diameter. HF wet etching does not control the final waist diameter to better than a few micrometers, and small diameter differences alter the evanescent-field overlap with the surrounding medium, the modal content of the waist, and the input/output coupling efficiency, which in turn shift the optimal sensing wavelength and the binding parameters extracted from the Langmuir fit. The limited diameter reproducibility of HF etching is therefore a key limitation of the present fabrication. Per-fiber normalization places fibers of differing absolute amplitude on a common 0–100% scale, but the underlying diameter variation still propagates into the per-fiber Kd and LOD and into the width of the cohort error bars. Tighter diameter control, for example, real-time transmission-monitored etching would be expected to reduce this variability and narrow the cohort spread.

A second contribution to the inter-fiber spread, in particular in the apparent Kd, is the fiber-to-fiber variation in the surface density of the immobilized capture antibody: functionalization is performed manually on each fiber, and differences in silanol activation and antibody coverage alter the effective avidity of the sensing surface, shifting the apparent dissociation constant away from the intrinsic solution-phase affinity of the antibody. Two complementary strategies mitigate this variability. Per-fiber wavelength calibration recovers each fiber’s own optimum sensing wavelength despite the diameter-driven shift, in software at no hardware or throughput cost (quantified below); it does not remove the residual inter-fiber spread in response amplitude, Kd and LOD, which is why the calibration is reported as a cohort of three fibers. Real-time transmission-monitored etching terminates the etch at a target spectral signature rather than a fixed time and thereby tightens the diameter distribution directly.

As an independent check on the wavelength-selection procedure, the analysis was repeated with the sensing wavelength defined without any use of the concentration-response data. For each PBS fiber, the wavelength was obtained from that fiber’s own pre-functionalization RI calibration as the wavelength of maximum bulk-RI sensitivity, and the MMP-8 dose–response was then evaluated at this predefined wavelength. The resulting cohort parameters are nearly unchanged: Kd = 7.4 versus 7.1 ng/mL and LOD = 0.040 versus 0.043 ng/mL, with the fit quality essentially unchanged and per-fiber LODs of 0.025–0.079 ng/mL. The sub-ng/mL detection limits reported in this work therefore do not depend on optimizing the sensing wavelength against the calibration data. Referencing the readout to each sensor’s own spectrum is also standard practice for etched and tapered fiber biosensors, which are typically interrogated by tracking a resonance or interference dip specific to each sensor [14,15,16,19,33,34].

### 3.3. Sensor Specificity and Antibody-Immobilization Validation

**Figure 5:**
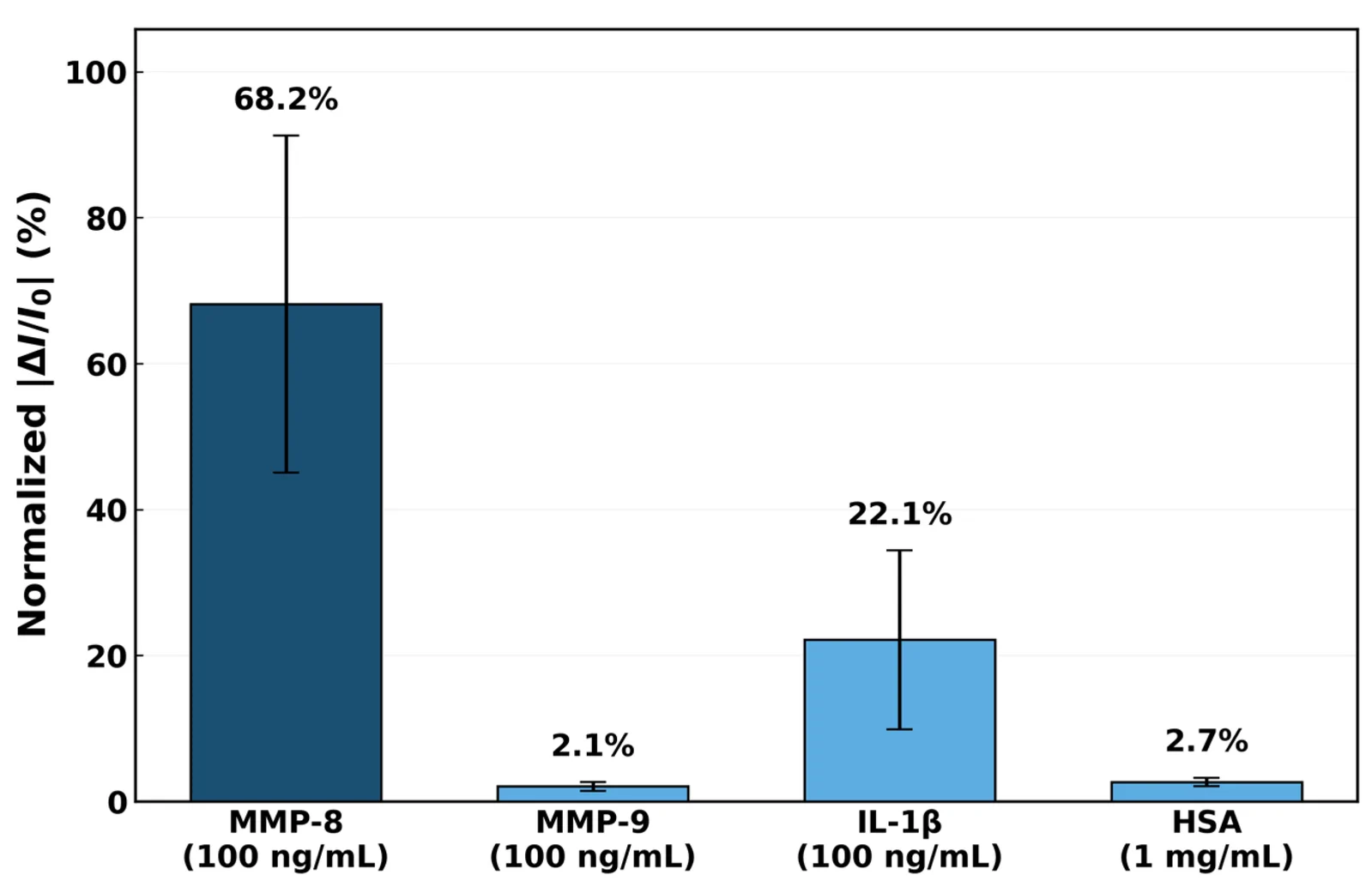
Normalized intensity response of the target MMP-8 (100 ng/mL) versus three non-target proteins: the structurally related protease MMP-9 (100 ng/mL), the inflammatory cytokine IL-1β (100 ng/mL), and human serum albumin (HSA, 1 mg/mL). Bars are the cohort mean ± SEM across three independently functionalized fibers.

The analytical specificity of the anti-MMP-8 functionalized sensor was evaluated against three non-target proteins spanning the principal interference modes relevant to oral fluid: a structurally related protease (MMP-9), a co-occurring inflammatory cytokine (IL-1β), and the most abundant interfering protein in saliva and serum (HSA). Each non-target was tested on three independently functionalized fibers using the post-rinse protocol, and the response was compared with the MMP-8 dose–response amplitude at the matched concentration (100 ng/mL). The MMP-8 target produced a strong response (68.2%), whereas the structurally related protease MMP-9 (2.1%) and the abundant interferent HSA, challenged at 1 mg/mL (2.7%), both gave negligible signals well below the target (Figure 5). The sensor therefore effectively rejects the two most diagnostically important interferents, the nearest structural homolog and bulk albumin. The inflammatory cytokine IL-1β gave a larger and more variable response (22.1%), indicating a partial cross-response. This difference is not explained by molecular size, since the larger MMP-9 (~92 kDa) and HSA (~66 kDa) would produce a greater signal per adsorbed molecule than the 17 kDa IL-1β [35,36,37]; it is consistent with surface affinity. MMP-9 is an acidic (pI ≈ 5.7), heavily glycosylated protein and HSA is acidic (pI ≈ 4.7) [36,37,38], so at pH 7.4, both carry a net negative charge, as does the BSA-blocked surface (BSA pI ≈ 4.7), and albumin adsorbs poorly onto an albumin-covered surface; electrostatic repulsion and the hydration shell of glycosylation therefore limit their non-specific adhesion [39], and neither carries an epitope for the anti-MMP-8 antibody. IL-1β, in contrast, is a small, near-neutral (pI ≈ 7), non-glycosylated cytokine [35,38] that can occupy defects in the BSA layer and residual reactive sites of the aminosilane–glutaraldehyde surface, and a fraction of it survives the PBS rinse; the strong fiber-to-fiber variability of its response is consistent with such non-specific adsorption rather than a reproducible recognition event [39]. Optimization of the antifouling surface, for example with casein- or PEG-based blocking, and its evaluation by challenging the sensor with non-target proteins over a concentration range before and after the improved blocking, is a separate study worth exploring in future work.

**Figure 6:**
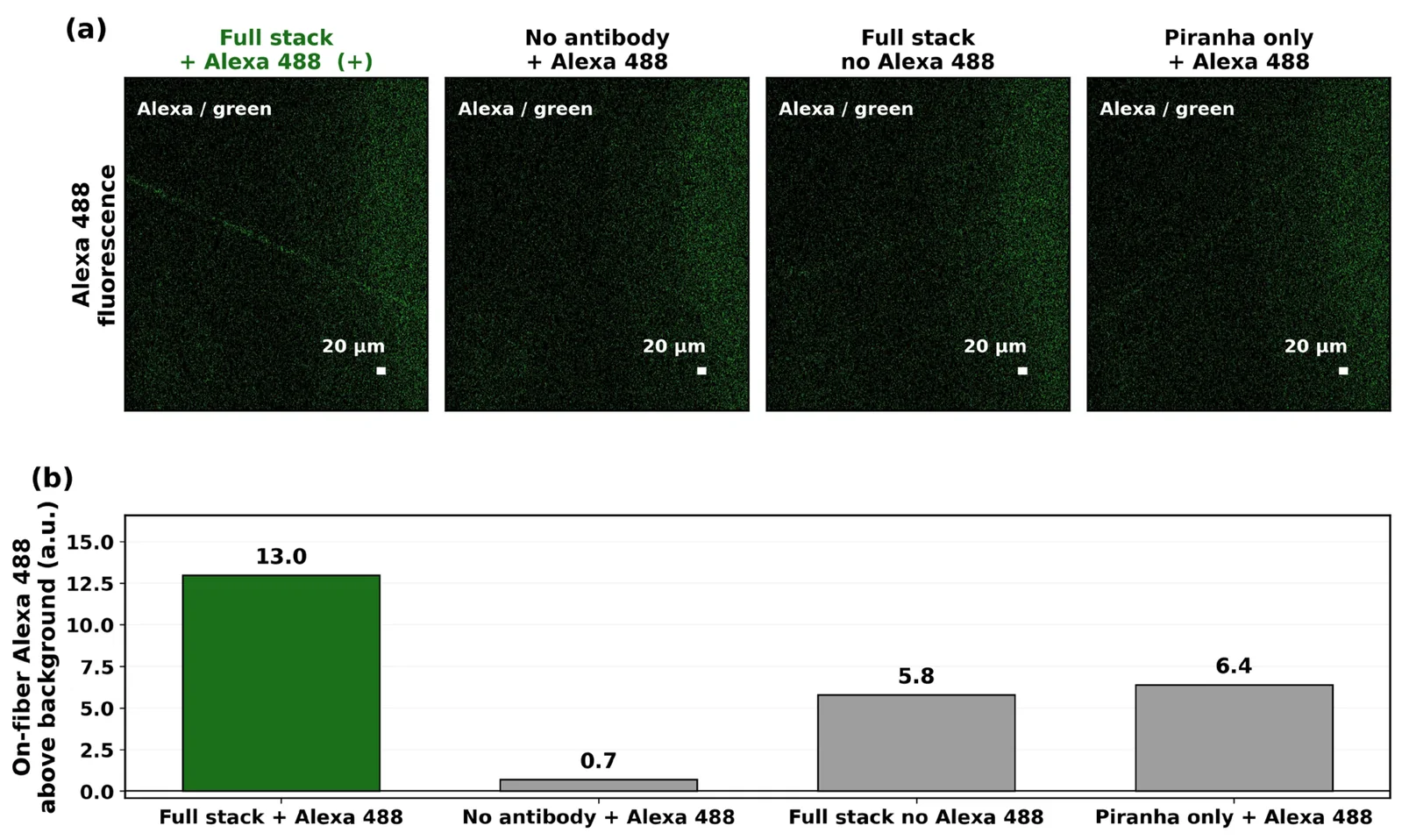
(**a**) Representative Alexa-488 (green) fluorescence images of the fiber surface for four conditions: the complete immobilization stack + Alexa 488 (positive), and three controls, no antibody + Alexa 488, complete stack without Alexa 488, and piranha-cleaned fiber + Alexa 488. (**b**) On-fiber Alexa-488 fluorescence, quantified as the mean intensity in a fixed-width band along the fiber centerline minus the local background (Section 2.10), in relative units (a.u.).

To verify that the captured signal originates from correctly immobilized capture antibody, the immobilization stack was labeled with an Alexa Fluor 488 anti-mouse secondary antibody and imaged by confocal microscopy (Figure S1, full DAPI/Alexa/overlay channels; Figure 6a, representative Alexa-488 panels). On-fiber Alexa-488 fluorescence, quantified along the fiber centerline above the local background (Figure 6b), was strong only for the complete stack (13.0 a.u.) and near zero for the no-antibody control (0.7 a.u.), an ~18-fold difference, demonstrating that secondary-antibody binding, and therefore the labeled signal, is antibody dependent. The no-dye (5.8 a.u.) and piranha-only (6.4 a.u.) controls showed only faint fiber background attributable to scatter/autofluorescence rather than specific dye capture. Together with the specificity data, this confirms that the sensor response is dominated by specific, antibody-mediated MMP-8 recognition.

### 3.4. MMP-8 Detection in Spiked Saliva

**Figure 7:**
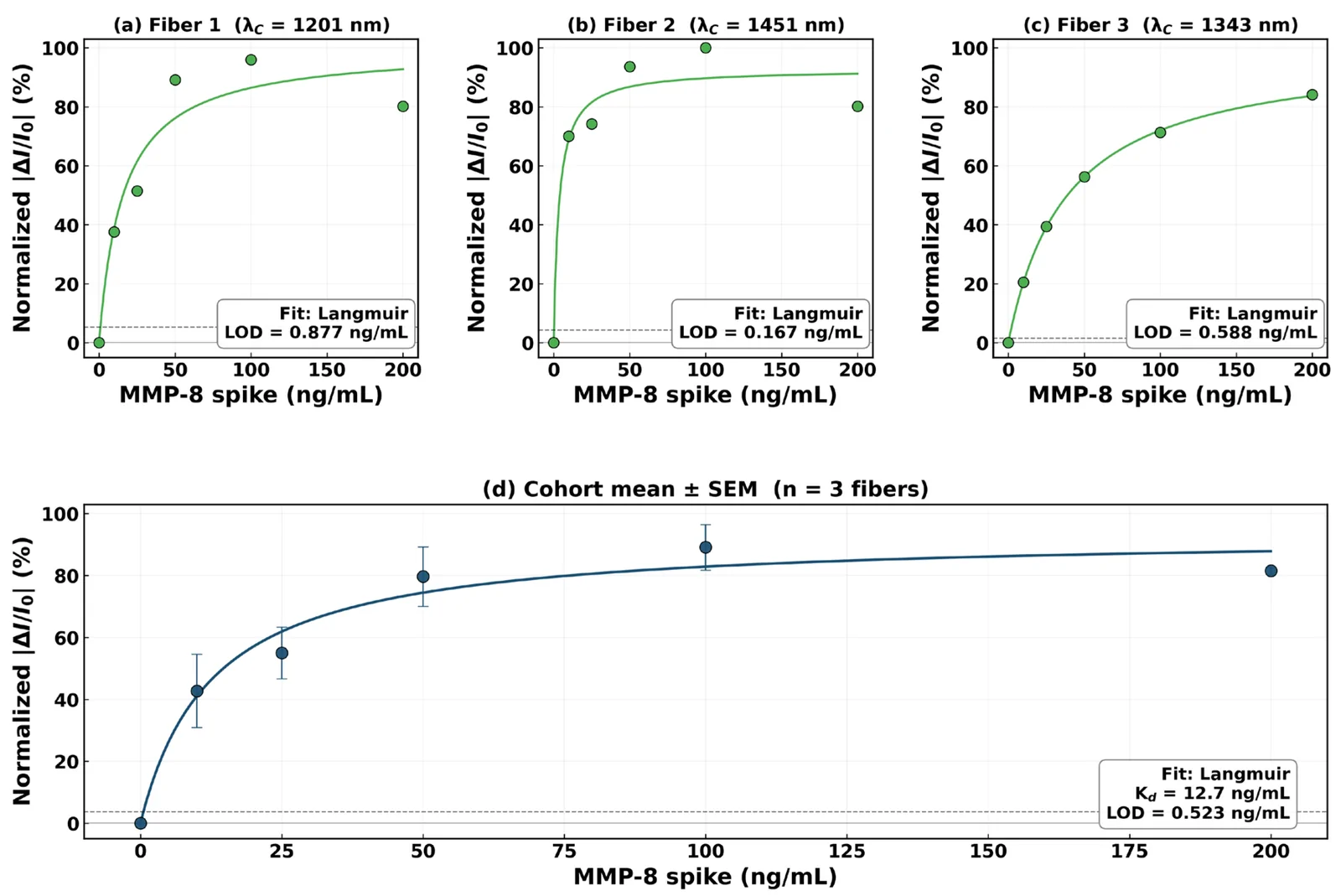
(**a**–**c**) Normalized intensity response of three individual antibody-functionalized fibers to increasing MMP-8 spike concentration at their selected sensing wavelengths (1201, 1451, and 1343 nm); responses were fitted with a Langmuir binding model, giving individual LODs of 0.877, 0.167, and 0.588 ng/mL, respectively. The dashed line marks the 3σ detection threshold. (**d**) Cohort response (mean ± SEM, n = 3 fibers) with a Langmuir fit yielding Kd = 12.7 ng/mL and an overall LOD of 0.52 ng/mL.

To assess the performance of the biosensor in a clinically relevant matrix, a cohort of three independently fabricated and functionalized fibers (n = 3) was tested with MMP-8 spiked into saliva over 0–200 ng/mL. The post-rinse, matrix-corrected response increased monotonically with the spike concentration and approached saturation, consistent with specific antibody–antigen binding described by a Langmuir isotherm (Figure 7). The cohort Langmuir fit (mean ± SEM, n = 3) gave an apparent Kd = 12.7 ng/mL and a cohort LOD of 0.52 ng/mL, approximately 38 times below the 20 ng/mL clinical threshold for periodontitis. Per-fiber LODs ranged from 0.167 to 0.877 ng/mL.

As expected, the LOD was lower in PBS (0.043 ng/mL) than in saliva (0.52 ng/mL). PBS is a simple buffer with no interfering molecules, so the sensor reaches its best sensitivity, whereas saliva contains additional molecules that raise the background and increase non-specific interactions, giving a higher LOD. This trend, a higher LOD in a complex biological matrix than in buffer, is well documented for optical and immunoassay biosensors, where matrix components and non-specific adsorption raise the background and can suppress specific binding, degrading the detection limit relative to buffered standards [40,41]. Importantly, the antibody–antigen binding itself is preserved across both matrices: the apparent dissociation constants are of the same order (K_d = 7.1 ng/mL in PBS and 12.7 ng/mL in saliva), both within the published affinity range for monoclonal anti-MMP-8 antibodies (1–100 ng/mL) [6].

The higher LOD in saliva has two measured origins: a higher apparent Kd (7.1 versus 12.7 ng/mL) and a larger ratio of baseline noise-to-saturation amplitude, the second being the dominant contribution. The higher apparent Kd in saliva reflects several concurrent mechanisms: hindered diffusion of MMP-8 through the viscous, mucin-rich fluid within the fixed 15 min incubation, which lowers the surface occupancy reached at a given concentration and raises the apparent, kinetically limited dissociation constant [42,43]; competitive adsorption of abundant salivary proteins (albumin, amylase, mucins) that partially occludes antibody paratopes [41,44]; sequestration of part of the spiked MMP-8 by endogenous inhibitors such as TIMP-1 and α2-macroglobulin [45,46], which the per-fiber blank subtraction does not remove; and small differences in pH and ionic strength that modify the electrostatics of the binding interface [27]. The larger noise term arises because the amount of residually bound matrix material that survives the rinse varies from exposure to exposure, which raises the effective baseline σ relative to the specific saturation amplitude [40,41].

All incubations in this work were performed at room temperature. Temperature would in principle affect the antibody–antigen affinity, the refractive index of the medium, and the rates of both specific binding and non-specific adsorption. The assay is, however, performed ex vivo: the collected saliva equilibrates to ambient temperature within minutes, so by the time of incubation and measurement, the sample is at room temperature regardless of its original in-body temperature, as will equally be the case in point-of-care use. The room-temperature characterization therefore matches the deployment condition of the sensor. Moreover, a static temperature offset cancels in the ΔI/I_0_ readout, since each response is referenced to a baseline acquired in the same matrix at the same temperature, and temperature fluctuations during a measurement are contained in the measured baseline σ.

To move beyond the blank-subtracted matrix-effect experiment toward quantitative performance, a spike-recovery analysis was performed on the saliva series as defined in analytical method validation [47,48]: each spike was back-calculated from the cohort and expressed as a percentage of the nominal spike. Because the calibration was fitted to the same spike series, this is a self-consistency check of quantitative readout rather than a validation against an independent sample set. The recovered concentrations were 10.7 ng/mL at a 10 ng/mL spike (107% recovery) and 18.3 ng/mL at a 25 ng/mL spike (73% recovery), within or close to the 80–120% acceptance range customary for immunoassays [48], while at and above 50 ng/mL the inversion becomes inaccurate (148% at 50 ng/mL, with larger deviations above) because the responses approach the Langmuir saturation plateau, where the calibration flattens and small signal differences translate into large concentration differences. Quantitative concentration readout is therefore accurate in the decision-relevant range around the 20 ng/mL clinical threshold, whereas at higher concentrations, the sensor operates as a threshold classifier rather than a proportional assay. These results were obtained on a single pooled-saliva source; spike recovery across saliva from multiple donors against an independent calibration, together with comparison against a reference immunoassay, is part of the planned clinical-sample validation.

### 3.5. Cross-Validation Against ELISA in Spiked Saliva

**Figure 8:**
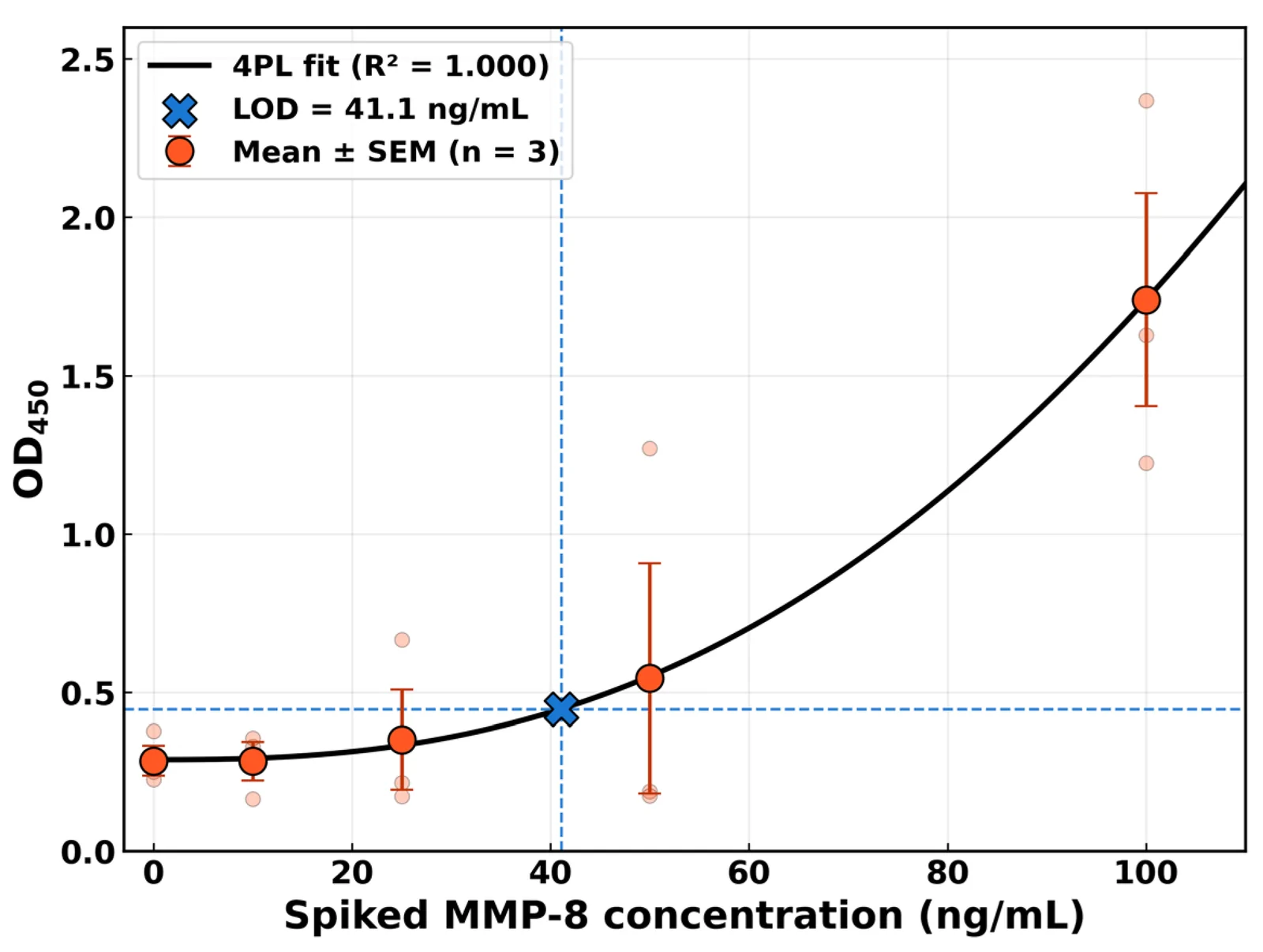
ELISA cross-validation in spiked saliva. Triplicate OD_450_ (mean ± SEM, n = 3 wells per concentration) for MMP-8 spiked into saliva at 0, 10, 25, 50, and 100 ng/mL. The dashed blue lines mark the kit-defined LOD threshold at OD_450_ = 0.45 (mean blank + 2 × SD), which the fit crosses at a spike concentration of 41.1 ng/mL.

To benchmark the fiber-optic biosensor against the gold-standard immunoassay in the same matrix, the MMP-8/saliva spike series (0, 10, 25, 50, 100 ng/mL) was assayed in triplicate on a commercial sandwich ELISA (Invitrogen EHMMP8) following the manufacturer’s protocol. The 4PL fit to the mean OD_450_ values gave a kit-defined LOD of 41.1 ng/mL, taken as the spike concentration at which the fit crosses the blank-based threshold OD_450_ = 0.45 (mean blank + 2 × SD) (Figure 8).

The measured saliva spike series ELISA LOD (41.1 ng/mL) is well above the kit’s stated buffer specification (~0.063 ng/mL functional sensitivity in the proprietary assay diluent) and above the ~0.09–0.16 ng/mL sensitivity typical of commercial total-MMP-8 ELISA kits measured against buffered standards [22]. This degradation is consistent with the well-documented loss of immunoassay sensitivity in complex fluids: firstly, matrix components (mucins, salts, and other additives) interfere with the capture-detection antibody pair and raise the assay background; and secondly, the triplicate wells showed high inter-replicate variability (for example, OD_450_ values spanning 0.19–1.27 at 50 ng/mL and 1.22–2.37 at 100 ng/mL), which inflates both the blank standard deviation and the fitted threshold. Antibody-based assays are known to display wide variability in quantifying MMP-8 in saliva, and commercial kits originally validated for serum or plasma are acknowledged to be affected when applied directly to the saliva matrix [11,49].

### 3.6. Comparison with Previously Reported MMP-8 Biosensors

The cohort LODs reported here (0.043 ng/mL in PBS, 0.52 ng/mL in spiked saliva) are, to the best of our knowledge, the lowest for a label-free MMP-8 biosensor, but sensitivity is not the metric that separates these platforms. With a clinical decision threshold of 20 ng/mL and advanced disease presenting at 100–200 ng/mL [8,9], the requirement is to resolve roughly one decade above 20 ng/mL, and every platform in Table 1 except the SAW immunosensor already clears that threshold by at least an order of magnitude. The sub-ng/mL LODs achieved here are therefore best understood as headroom rather than as a requirement: they place the clinically relevant window in the well-resolved portion of the dose–response and leave margin for the additional signal loss expected on moving from spiked to clinical saliva.

The transduction fundamentals of the platform, HF etching to expose the evanescent field of the guided mode and covalent antibody immobilization through APTES–glutaraldehyde chemistry, are shared with previously reported etched-fiber biosensors [14,15,26]. Comparable optical-fiber sensors have been characterized under conditions that differ from the present work in the matrix, the signal acquisition, or the detection threshold: the saliva is diluted 1:50 in PBS [19,29,50,51] or filtered in-line before reaching the sensor [40], and grating-based evanescent sensors have been characterized in buffer only [14,15,17]; the response is recorded with the sensor immersed in the sample [14,15,17,40,52]; and the detection threshold is derived from blank or baseline noise measured in buffer rather than in the matrix [14,15,40,50,51]. What is specific to this work is the measurement methodology that makes these fundamentals quantitative in undiluted saliva. Every quantification spectrum is acquired in PBS after incubation and rinsing, so that the bulk refractive index and scattering of whole saliva, which otherwise dominate the in-chamber signal, are excluded and only the surface-bound analyte fraction is read. A per-fiber matrix subtraction referenced to an unspiked saliva blank cancels endogenous MMP-8 and residual non-specific matrix binding on a per-sensor basis. Per-fiber selection of the sensing wavelength accounts for the fiber-to-fiber shift of the optimal wavelength caused by the diameter tolerance of HF wet etching, and the detection limit is determined against baseline noise measured in the matched matrix rather than in buffer, following the recommendation to account for biological noise in real samples [41]. Together, these steps preserve the specific binding amplitude while suppressing the matrix background, and account for the sub-ng/mL LOD retained in spiked saliva (0.52 ng/mL) despite the complexity of the matrix.

What does separate these platforms is what each costs to produce. Every prior device in Table 1 depends on at least one process that does not scale cheaply, vacuum metal deposition, nanomaterial synthesis and electrode modification, lithographic layer definition, or enzymatic amplification with its associated reagent cold chain. The sensor reported here requires none of these: fabrication reduces to a single wet-etch on stock telecommunications fiber, carried out in parallel in a multi-fiber holder without heat or vacuum equipment, making a low-cost disposable consumable plausible. Two limitations qualify this. First, HF etching does not reproduce the waist diameter to better than a few micrometers, and this, rather than process complexity, is the real barrier to manufacturing consistency; closed-loop transmission-monitored etching would be needed to address it. Second, unlike the SAW device, which was evaluated on saliva from 177 subjects [20], the present sensor has been characterized only in spiked matrices and awaits validation on clinical samples. Third, the present study used three fibers per condition; this cohort size demonstrates proof-of-concept analytical performance and reveals the fabrication variability discussed above, but establishing manufacturing reproducibility will require substantially larger fabrication batches, which the transmission-monitored etching described in Section 3.2 is designed to enable.

## 4. Conclusions

This work shows that a label-free MMP-8 biosensor with clinically sufficient performance can be built without any of the fabrication steps that current platforms depend on. A single wet-etch of stock telecommunications fiber, with no metal coating, nanomaterial, lithography, or label, gave sub-ng/mL detection limits in both PBS and spiked saliva, more than an order of magnitude below the 20 ng/mL periodontitis threshold, across a dynamic range covering the full clinical window through advanced disease. Benchmarked on the same spike series in the same matrix, the sensor detected MMP-8 nearly two orders of magnitude below a commercial sandwich ELISA, indicating that the sensitivity loss immunoassays suffer in saliva reflects the assay format rather than the matrix itself. Because the sensing element is a single etched length of stock fiber, read out with a broadband source and a compact spectrometer, the platform is well suited to production as a low-cost disposable. Tighter control of the etched-waist diameter, improved surface blocking, and validation on clinical saliva from periodontitis patients will consolidate this into a practical test for periodontal disease. For deployment, the sensing wavelength of every fiber should be predefined from its own refractive-index calibration performed before functionalization, using the wavelength of maximum refractive-index sensitivity, and each fiber should then be used for a single measurement at one concentration, so that the wavelength choice is fixed before, and independently of, the analytical calibration and every calibration point comes from an independent sensor.

## Data Availability

The data presented in this study are available on request from the corresponding author.

## Supplementary Materials

The following supporting information can be downloaded at: https://www.mdpi.com/article/10.3390/bios16090491/s1, Figure S1: Confocal immunofluorescence validation of antibody immobilization on the etched-fiber surface, showing the full DAPI, Alexa-488, and overlay channels for the complete immobilization stack and the three controls.

## Abbreviations

The following abbreviations are used in this manuscript:

APTES	(3-aminopropyl)triethoxysilane
BSA	bovine serum albumin
DI	deionized
ELISA	enzyme-linked immunosorbent assay
GCF	gingival crevicular fluid
HF	hydrofluoric acid
HSA	human serum albumin
IL-1β	interleukin-1 beta
LOD	limit of detection
MMP-8	matrix metalloproteinase-8
MMP-9	matrix metalloproteinase-9
PBS	phosphate-buffered saline
RI	refractive index
SAW	surface acoustic wave
SEM	standard error of the mean
SMF	single-mode fiber
SPR	surface plasmon resonance

## References

[1] Eke P.I., Dye B.A., Wei L., Slade G.D., Thornton-Evans G.O., Borgnakke W.S., Taylor G.W., Page R.C., Beck J.D., Genco R.J. (2015). Update on prevalence of periodontitis in adults in the United States: NHANES 2009 to 2012. J. Periodontol..

[2] Kassebaum N.J., Bernabé E., Dahiya M., Bhandari B., Murray C.J.L., Marcenes W. (2014). Global burden of severe periodontitis in 1990–2010: A systematic review and meta-regression. J. Dent. Res..

[3] Sanz M., Marco del Castillo A., Jepsen S., Gonzalez-Juanatey J.R., D’Aiuto F., Bouchard P., Chapple I., Dietrich T., Gotsman I., Graziani F. (2020). Periodontitis and cardiovascular diseases: Consensus report. J. Clin. Periodontol..

[4] Tonetti M.S., Jepsen S., Jin L., Otomo-Corgel J. (2017). Impact of the global burden of periodontal diseases on health, nutrition and wellbeing of mankind: A call for global action. J. Clin. Periodontol..

[5] Loesche W.J., Grossman N.S. (2001). Periodontal disease as a specific, albeit chronic, infection: Diagnosis and treatment. Clin. Microbiol. Rev..

[6] Sorsa T., Gursoy U.K., Nwhator S., Hernández M., Tervahartiala T., Leppilahti J., Gursoy M., Könönen E., Emingil G., Pussinen P.J. (2016). Analysis of matrix metalloproteinases, especially MMP-8, in gingival crevicular fluid, mouthrinse and saliva for monitoring periodontal diseases. Periodontology 2000.

[7] Luchian I., Goriuc A., Sandu D., Covasa M. (2022). The role of matrix metalloproteinases (MMP-8, MMP-9, MMP-13) in periodontal and peri-implant pathological processes. Int. J. Mol. Sci..

[8] Hernández M., Dutzan N., García-Sesnich J., Abusleme L., Dezerega A., Silva N., González F.E., Vernal R., Sorsa T., Gamonal J. (2011). Host-pathogen interactions in progressive chronic periodontitis. J. Dent. Res..

[9] Sorsa T., Alassiri S., Grigoriadis A., Räisänen I.T., Pärnänen P., Nwhator S.O., Gieselmann D.R., Sakellari D. (2020). Active MMP-8 (aMMP-8) as a grading and staging biomarker in the periodontitis classification. Diagnostics.

[10] Leppilahti J.M., Hernandez-Rios P.A., Gamonal J.A., Tervahartiala T., Brignardello-Petersen R., Mäntylä P., Sorsa T., Hernández M. (2014). Matrix metalloproteinases and myeloperoxidase in gingival crevicular fluid provide site-specific diagnostic value for chronic periodontitis. J. Clin. Periodontol..

[11] Umeizudike K.A., Lähteenmäki H., Räisänen I.T., Taylor J.J., Preshaw P.M., Bissett S.M., Tervahartiala T., Nwhator S.O., Pärnänen P., Sorsa T. (2022). Ability of matrix metalloproteinase-8 biosensor, IFMA, and ELISA immunoassays to differentiate between periodontal health, gingivitis, and periodontitis. J. Periodontal Res..

[12] Yin M.-J., Gu B., An Q.-F., Yang C., Guan Y.L., Yong K.-T. (2018). Recent development of fiber-optic chemical sensors and biosensors: Mechanisms, materials, micro/nano-fabrications and applications. Coord. Chem. Rev..

[13] Caucheteur C., Guo T., Albert J. (2015). Review of plasmonic fiber optic biochemical sensors: Improving the limit of detection. Anal. Bioanal. Chem..

[14] Sypabekova M., Korganbayev S., González-Vila Á., Caucheteur C., Shaimerdenova M., Ayupova T., Bekmurzayeva A., Vangelista L., Tosi D. (2019). Functionalized etched tilted fiber Bragg grating aptasensor for label-free protein detection. Biosens. Bioelectron..

[15] Bekmurzayeva A., Dukenbayev K., Shaimerdenova M., Bekniyazov I., Ayupova T., Sypabekova M., Molardi C., Tosi D. (2018). Etched fiber Bragg grating biosensor functionalized with aptamers for detection of thrombin. Sensors.

[16] Tian Y., Wang W., Wu N., Zou X., Wang X. (2011). Tapered optical fiber sensor for label-free detection of biomolecules. Sensors.

[17] Loyez M., Albert J., Caucheteur C., Wattiez R. (2018). Cytokeratins biosensing using tilted fiber gratings. Biosensors.

[18] Spierings G.A.C.M. (1993). Wet chemical etching of silicate glasses in hydrofluoric acid based solutions. J. Mater. Sci..

[19] Guida L., Bencivenga D., Annunziata M., Arcadio F., Borriello A., Della Ragione F., Formisano A., Piccirillo A., Zeni L., Cennamo N. (2023). An optical fiber-based point-of-care test for periodontal MMP-8 detection: A proof of concept. J. Dent..

[20] Taylor J.J., Jaedicke K.M., van de Merwe R.C., Bissett S.M., Landsdowne N., Whall K.M., Pickering K., Thornton V., Lawson V., Yatsuda H. (2019). A prototype antibody-based biosensor for measurement of salivary MMP-8 in periodontitis using surface acoustic wave technology. Sci. Rep..

[21] Tortolini C., Gigli V., Angeloni A., Tasca F., Thanh N.T.K., Antiochia R. (2024). A disposable immunosensor for the detection of salivary MMP-8 as biomarker of periodontitis. Bioelectrochemistry.

[22] Wu Z., Wang Y., Zhang Y., Yi J., Li Y., Wang J., Hu M., Wang D. (2024). Ultrasensitive detection of MMP-8 in saliva for monitoring periodontitis using an Immuno-CRISPR/Cas12a assay. Sens. Actuators B Chem..

[23] Lako A., Sypabekova M. (2025). Tapered Optical Fiber Optofluidics: Bridging In-Fiber and Outside-Fiber Architectures Toward Autonomous Lab-on-Fiber Biosensing. Sensors.

[24] Sorsa T., Nwhator S.O., Sakellari D., Grigoriadis A., Umeizudike K.A., Brandt E., Keskin M., Tervahartiala T., Pärnänen P., Gupta S. (2022). aMMP-8 oral fluid PoC test in relation to oral and systemic diseases. Front. Oral Health.

[25] Lide D.R. (2004). CRC Handbook of Chemistry and Physics.

[26] Sypabekova M., Aitkulov A., Blanc W., Tosi D. (2020). Reflector-less nanoparticles doped optical fiber biosensor for the detection of proteins: Case thrombin. Biosens. Bioelectron..

[27] Reverberi R., Reverberi L. (2007). Factors affecting the antigen–antibody reaction. Blood Transfus..

[28] Goode J.A., Rushworth J.V.H., Millner P.A. (2015). Biosensor regeneration: A review of common techniques and outcomes. Langmuir.

[29] Annunziata M., Arcadio F., Borriello A., Bencivenga D., Piccirillo A., Stampone E., Zeni L., Cennamo N., Della Ragione F., Guida L. (2023). A novel plasmonic optical-fiber-based point-of-care test for periodontal MIP-1α detection. iScience.

[30] Currie L.A. (1999). Detection and quantification limits: Origins and historical overview. Anal. Chim. Acta.

[31] Villatoro J., Monzón-Hernández D., Mejía E. (2003). Fabrication and modeling of uniform-waist single-mode tapered optical fiber sensors. Appl. Opt..

[32] Vörös J. (2004). The density and refractive index of adsorbing protein layers. Biophys. J..

[33] Socorro A.B., Santamaría E., Fernández-Irigoyen J., Del Villar I., Corres J.M., Arregui F.J., Matias I.R. (2017). Fiber-optic immunosensor based on an etched SMS structure. IEEE J. Sel. Top. Quantum Electron..

[34] Bekmurzayeva A., Nurlankyzy M., Abdossova A., Myrkhiyeva Z., Tosi D. (2024). All-fiber label-free optical fiber biosensors: From modern technologies to current applications. Biomed. Opt. Express.

[35] Dinarello C.A. (2011). Interleukin-1 in the pathogenesis and treatment of inflammatory diseases. Blood.

[36] Vandooren J., Van den Steen P.E., Opdenakker G. (2013). Biochemistry and molecular biology of gelatinase B or matrix metalloproteinase-9 (MMP-9): The next decade. Crit. Rev. Biochem. Mol. Biol..

[37] Fanali G., di Masi A., Trezza V., Marino M., Fasano M., Ascenzi P. (2012). Human serum albumin: From bench to bedside. Mol. Asp. Med..

[38] The UniProt Consortium (2023). UniProt: The Universal Protein Knowledgebase in 2023. Nucleic Acids Res..

[39] Rabe M., Verdes D., Seeger S. (2011). Understanding protein adsorption phenomena at solid surfaces. Adv. Colloid Interface Sci..

[40] Stevens R.C., Soelberg S.D., Near S., Furlong C.E. (2008). Detection of cortisol in saliva with a flow-filtered, portable surface plasmon resonance biosensor system. Anal. Chem..

[41] Masson J.-F. (2020). Consideration of sample matrix effects and “biological” noise in optimizing the limit of detection of biosensors. ACS Sens..

[42] Schipper R.G., Silletti E., Vingerhoeds M.H. (2007). Saliva as research material: Biochemical, physicochemical and practical aspects. Arch. Oral Biol..

[43] Squires T.M., Messinger R.J., Manalis S.R. (2008). Making it stick: Convection, reaction and diffusion in surface-based biosensors. Nat. Biotechnol..

[44] Blaszykowski C., Sheikh S., Thompson M. (2012). Surface chemistry to minimize fouling from blood-based fluids. Chem. Soc. Rev..

[45] Sorsa T., Tjäderhane L., Konttinen Y.T., Lauhio A., Salo T., Lee H.-M., Golub L.M., Brown D.L., Mäntylä P. (2006). Matrix metalloproteinases: Contribution to pathogenesis, diagnosis and treatment of periodontal inflammation. Ann. Med..

[46] Gursoy U.K., Könönen E., Pradhan-Palikhe P., Tervahartiala T., Pussinen P.J., Suominen-Taipale L., Sorsa T. (2010). Salivary MMP-8, TIMP-1, and ICTP as markers of advanced periodontitis. J. Clin. Periodontol..

[47] Magnusson B., Örnemark U. (2014). Eurachem Guide: The Fitness for Purpose of Analytical Methods—A Laboratory Guide to Method Validation and Related Topics.

[48] International Council for Harmonisation (2023). ICH Harmonised Guideline Q2(R2): Validation of Analytical Procedures.

[49] Boynes S.G., Sofiyeva N., Saw T., Nieto V., Palomo L. (2025). Assessment of salivary matrix metalloproteinase (MMP8) and activated salivary matrix metalloproteinase (aMMP8) in periodontitis patients: A systematic review and meta-analysis. Front. Oral Health.

[50] Cennamo N., Bencivenga D., Annunziata M., Arcadio F., Stampone E., Piccirillo A., Della Ragione F., Zeni L., Guida L., Borriello A. (2024). Plasmon resonance biosensor for interleukin-1β point-of-care determination: A tool for early periodontitis diagnosis. iScience.

[51] Arcadio F., Seggio M., Pitruzzella R., Zeni L., Bossi A.M., Cennamo N. (2024). An efficient bio-receptor layer combined with a plasmonic plastic optical fiber probe for cortisol detection in saliva. Biosensors.

[52] Rashidova G., Tilegen M., Pham T.T., Bekmurzayeva A., Tosi D. (2024). Functionalized optical fiber ball-shaped biosensor for label-free, low-limit detection of IL-8 protein. Biomed. Opt. Express.

