# Supplementary material for: Periodontal Disease Diagnosis by a Chemically Etched Single-Mode Fiber-Optic Biosensor for Label-Free Detection of Matrix Metalloproteinase-8 (MMP-8)
Source: Biosensors (Basel). 2026 Sep 3;16(9):491. doi: 10.3390/bios16090491 (PMC13604195; doi:10.3390/bios16090491)
Supplement: Supplementary file 1 [file biosensors-16-00491-s001.zip › biosensors-4516112-supplementary.pdf]

biosensors

Article

Periodontal Disease Diagnosis by a Chemically Etched Single-Mode Fiber-Optic Biosensor for Label-Free Detection of Matrix Metalloproteinase-8 (MMP-8)

Rigoberto Tovar [1], Sarkis Sozkes [1,2] and Marzhan Sypabekova [1,*]

[1] Department of Bioengineering, Civil Engineering, and Environmental Engineering, U. A. Whitaker College of Engineering, Florida Gulf Coast University, 10501 FGCU Boulevard South, Fort Myers, FL 33965, USA; rtovar0243@eagle.fgcu.edu (R.T.); ssozkes@fgcu.edu (S.S.)
[2] Department of Biomaterials, Biomedical Engineering Corlu Faculty, Tekirdag Namik Kemal University, Yılmaz Alpaslan Cd. No:25, 59850 Corlu, 59860 Tekirdag, Turkey
* Correspondence: msypabekova@fgcu.edu

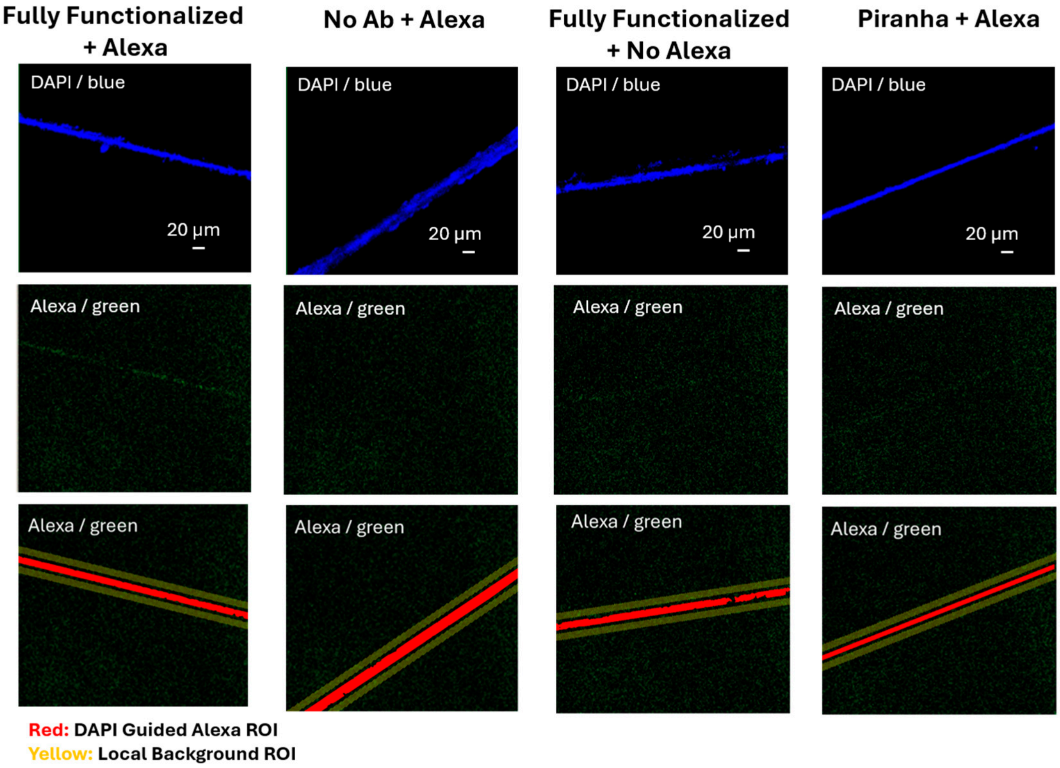

Figure S1. Confocal fluorescence validation of antibody immobilization on the fiber surface. Rows show the DAPI (blue), Alexa Fluor 488 (green), and overlay channels for the four conditions (complete immobilization stack, no-antibody, no-Alexa-dye, and piranha-only controls). In the overlay, the fiber centerline used for on-fiber signal quantification is highlighted in red with the flanking background bands in yellow. Strong on-fiber Alexa-488 signal appears only for the complete stack, confirming antibody-dependent labeling; the corresponding quantification is shown in main-text Figure 4b. Scale bars, 20 µm.

 https://doi.org/10.3390/bios16090491
